# An Improved RD Algorithm for Maneuvering Bistatic Forward-Looking SAR Imaging with a Fixed Transmitter

**DOI:** 10.3390/s17051152

**Published:** 2017-05-19

**Authors:** Yue Yuan, Si Chen, Huichang Zhao

**Affiliations:** School of Electronic & Optical Engineering, Nanjing University of Science & Technology, Nanjing 210094, China; 313104002284@njust.edu.cn (Y.Y.); zhaohch@njust.edu.cn (H.Z.)

**Keywords:** 2-D frequency spectrum, method of series reversion (MSR), SAR imaging, bistatic forward-looking SAR (BFL-SAR)

## Abstract

In order to improve the azimuth resolution beyond what monostatic synthetic aperture radar (SAR) can achieve in the forward-looking area, an asymmetric configuration bistatic SAR system and its imaging algorithm are proposed in this paper. The transmitter is mounted on a fixed platform in side-looking mode while the receiver moves along a nonlinear trajectory in forward-looking mode. Due to the high velocity and acceleration of the maneuvering platform in both along-track and height direction, the traditional algorithms are no longer applicable. In this paper, a new algorithm based on the high precise 2-D frequency spectrum is proposed, which takes high-order Taylor series expansion terms of the slant range into consideration. The proposed algorithm compensates high-order range-azimuth coupling terms to guarantee the focus accuracy in SAR imaging. The simulation results and error analysis validate the effectiveness of the proposed algorithm and the correctness of our analysis.

## 1. Introduction

Bistatic forward-looking synthetic aperture radar (BFL-SAR) has drawn much more attention because of its high resolution in both range and the azimuth direction, which fills in the blanks of conventional monostatic SAR. The BFL-SAR has many advantages, such as low probability interception, and immunity to physical attacks and electronic countermeasures, which is why it has significant potential in airplane navigation, missile guidance, and target detection.

Many studies have been made on BFL-SAR [1,2,3,4]. The ground moving target (GMT) detection and imaging theories were discussed for BFL-SAR in [5,6]. Chen introduced the general situation and the method for BFL-SAR [7]. In addition, several experiments have been carried out and the results demonstrate the feasibility of BFL-SAR [8,9]. Differing from monostatic SAR, the slant range in bistatic SAR has two hyperbolic functions, which are defined as double square root (DSR) terms [10], thus, the principle of the stationary phase (POSP) could not be directly used in BFL-SAR. To derive the 2-D frequency spectrum, some researchers transformed the DSR term into a single square root regarded as the monostatic configuration [11]. Raw data simulations for bistatic SAR were proposed in [12,13], which are based on their 2-D frequency spectra. However, the platforms are moving horizontally without acceleration and vertical speed. Huang introduced an advanced hyperbolic approximation method to accurately fit the cubic term of the range history [14]. Some researchers adopted hyperbolic equivalent methods with compensating variation to obtain the 2-D frequency spectrum [15,16]. The equivalence error cannot be ignored, especially in a high-speed maneuvering platform. Loffeld proposed a new method for 2-D frequency spectrum derivation [17]. The limitation of Loffeld’s formula is the reduced accuracy with a difference between two stationary points. In the application of missile terminal guidance and other maneuvering platforms, the platform moves with high velocities and accelerations [18]. The method of series reversion (MSR) can obtain the exact spectrum, theoretically [19,20]. The accuracy mainly depends on the order of the expansion terms. Zhang et al. used a high-order range equation and phase compensation to improve the accuracy of the range history and 2-D spectrum [21].

Based on previous research, this paper discusses an asymmetric bistatic configuration with a forward-looking receiver and a stationary transmitter, in which the slant range is kept up to its fourth-order Taylor series. Firstly, linear range walk correction (LRWC) operation is performed in the range frequency azimuth time domain. Secondly, the method of the series reversion and POSP are adopted in the derivation of 2-D frequency spectrum. Then the range compression, range cell migration (RCM), secondary range compression (SRC), and high-order range-azimuth coupling terms are compensated in the 2-D frequency domain. Finally, the azimuth compression is implemented by a matched filter in the range Doppler domain.

This paper is organized as follows: In Section 2, the geometry and signal models are established. In Section 3, the 2-D frequency spectrum is derived by MSR. Section 4 explores the proposed imaging algorithm for BFL-SAR. Section 5 shows the simulation results and error analysis of proposed algorithm. The conclusion of this letter is given in Section 6.

## 2. Geometry and Signal Model

**Figure 1:**
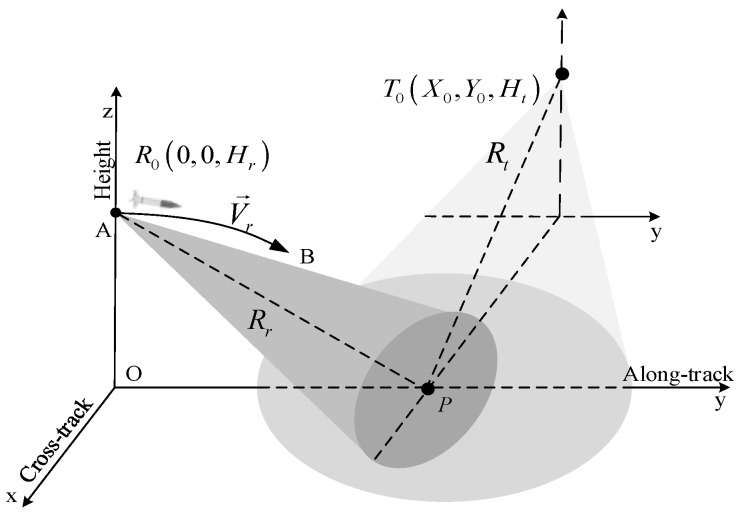
Geometry configuration of the MBFL-SAR.

The geometry configuration of BFL-SAR with a stationary transmitter and a maneuvering receiver is shown in Figure 1. The receiver works in forward-looking mode and travels in a descending curvilinear path along curve AB in plane yoz as the antenna points in the same direction. The fixed transmitter is mounted on a high tower where it can illuminate the imaging area easily. $t_{a}$ and $t_{r}$ denote the slow time (azimuth time) and the fast time (range time). The positions of the transmitter and receiver are $T_{0}\left(X_{t},Y_{t},H_{t}\right)$ and $R_{0}\left(0,0,H_{r}\right)$ at $t_{a}=0$. The initial velocity and acceleration of the receiver are $V_{0}=\left(0,V_{\mathrm{ry}0},V_{\mathrm{rz}0}\right)$ and $a=\left(0,a_{\mathrm{ry}},a_{\mathrm{rz}}\right)$, respectively. Therefore, the location of the receiver is $R\left(0,V_{\mathrm{ry}0}t_{a}+0.5a_{\mathrm{ry}}t_{a}^{2},H_{r}+V_{\mathrm{rz}0}t_{a}+0.5a_{\mathrm{rz}}t_{a}^{2}\right)$ at the time $t_{a}$, supposing the projection of receiver in xoy is the coordinates’ center O at $t_{a}=0$. $P\left(x_{p},y_{p},0\right)$ is an arbitrary ground point target in the imaging area. The instantaneous slant range from the transmitter and receiver to the target P at slow time $t_{a}$ are $R_{t}$ and $R_{r}$, respectively.

Supposing that the transmitted signal is the linear frequency modulation (LFM) signal [9], and the position of the point target P is $\left(x_{p},y_{p},0\right)$, then we have the instantaneous slant range of the high-speed maneuvering MBFL-SAR $R\left(t_{a}\right)$, which can be described by:

(1) $$\begin{array}{l} R\left(t_{a}\right)=R_{t}+R_{r}\left(t_{a}\right)=\sqrt{{\left(X_{t}-x_{p}\right)}^{2}+{\left(Y_{t}-y_{p}\right)}^{2}+{\left(H_{t}\right)}^{2}}\\ +\sqrt{x_{p}^{2}+{\left(V_{\mathrm{ry}0}t_{a}+\frac{1}{2}a_{\mathrm{ry}}t_{a}^{2}-y_{p}\right)}^{2}+{\left(H_{r}+V_{\mathrm{rz}0}t_{a}+\frac{1}{2}a_{\mathrm{rz}}t_{a}^{2}\right)}^{2}} \end{array}$$

where $R_{t}$ and $R_{r}\left(t_{a}\right)$ are the transmitter range and receiver range, respectively.

The signal model of the BFL-SAR is similar to the monostatic SAR, except for the range history. The transmitter range is only dependent on the position of P and independent of slow time $t_{a}$. Thus, the azimuth resolution depends on the motion of the receiver alone.

## 3. Derivation of 2-D Point Target Frequency Spectrum

Generally, we can obtain the 2-D frequency spectrum easily after the azimuth FFT. However, due to the existence of velocities and accelerations in both along-track and along-height directions, it is difficult to calculate the stationary point directly. Here, we expand the instantaneous slant range $R\left(t_{a}\right)$ into a Taylor series at $t_{a}=0$ as:

(2) $$R\left(t_{a}\right)=R_{t}+R_{r0}+\left(k_{1c}+k_{1}\right)t_{a}+k_{2}t_{a}^{2}+k_{3}t_{a}^{3}+k_{4}t_{a}^{4}+o\left(t_{a}^{4}\right)$$

where:

(3) $$R_{r0}=\sqrt{x_{p}^{2}+y_{p}^{2}+H_{r}^{2}}$$

(4) $$k_{1c}=\frac{-V_{\mathrm{ry}0}y_{\mathrm{pc}}+V_{\mathrm{rz}0}H_{r}}{R_{r0}}$$

(5) $$k_{1}=\frac{V_{\mathrm{ry}0}\left(y_{\mathrm{pc}}-y_{p}\right)}{R_{r0}}$$

(6) $$k_{2}=\frac{-a_{\mathrm{ry}}y_{p}+a_{\mathrm{rz}}H_{r}+{V_{\mathrm{ry}0}}^{2}+{V_{\mathrm{rz}0}}^{2}-{\left(k+k_{1c}\right)}^{2}}{2R_{r0}}$$

(7) $$k_{3}=\frac{V_{\mathrm{ry}0}a_{\mathrm{ry}}+V_{\mathrm{rz}0}a_{\mathrm{rz}}-2\left(k_{1}+k_{1c}\right)k_{2}}{2R_{r0}}$$

(8) $$k_{4}=\frac{{a_{\mathrm{ry}}}^{2}+{a_{\mathrm{rz}}}^{2}}{8R_{r0}}-\frac{2\left(k_{1}+k_{1c}\right)k_{3}+k_{2}^{2}}{2R_{r0}}$$

where $R_{r0}$ is the instantaneous range at $t_{a}=0$; $y_{\mathrm{pc}}$ is the y-axis of scene center point $P_{c}$; $k_{1c}$ is the linear coefficient at $P_{c}$ which represents the coefficient of the linear range walk (LRW); $k_{1}$ is the residual LRW coefficient of the target, which is far from the scene center; and $k_{2}$, $k_{3}$, and $k_{4}$ are the quadratic, cubic, and quartic coefficients, respectively. Here, the Taylor expansion series is kept up to fourth-order term.

In this bistatic configuration, we compensate the LRW at the scene center point $P_{c}$, so the LRWC function can be written as:

(9) $$H_{1}\left(f_{r},t_{a}\right)=\exp\left[j2\pi \frac{\left(f_{c}+f_{r}\right)}{c}k_{1c}t_{a}\right]$$

After LRWC, the signal can be written as:

(10) $$\begin{array}{l} s_{r3}\left(f_{r},t_{a}\right)=W_{r}\left(f_{r}\right)a_{a}\left(t_{a}\right)\cdot \exp\left(-j\pi \frac{f_{r}^{2}}{\gamma }\right)\\ \times \exp\left[-j2\pi \frac{\left(f_{c}+f_{r}\right)}{c}\left(R_{t}+R_{r0}+k_{1}t_{a}+k_{2}t_{a}^{2}+k_{3}t_{a}^{3}+k_{4}t_{a}^{4}\right)\right] \end{array}$$

Then, we can derive the 2-D frequency spectrum by the method of POSP and series reversion as:

(11) $$s_{r4}\left(f_{r},f_{a}\right)=W_{r}\left(f_{r}\right)W_{a}\left(f_{a}\right)\cdot \exp\left[j\psi \left(f_{r},f_{a}\right)\right]$$

where:

(12) $$\begin{array}{l} \psi \left(f_{r},f_{a}\right)=-\pi \frac{f_{r}^{2}}{\gamma }-2\pi \frac{R_{t}+R_{r0}}{A}+\frac{2\pi AA_{1}}{2}{\left(f_{a}+\frac{k_{1}}{A}\right)}^{2}\\ \;-2\pi \frac{A^{2}A_{2}}{3}{\left(f_{a}+\frac{k_{1}}{A}\right)}^{3}+2\pi \frac{A^{3}A_{3}}{4}{\left(f_{a}+\frac{k_{1}}{A}\right)}^{4} \end{array}$$

and:

(13) $$\{\begin{array}{cc} A=\frac{c}{f_{c}+f_{r}} & A_{1}=\frac{1}{2k_{2}}\\ A_{2}=-\frac{3k_{3}}{8k_{2}^{3}} & A_{3}=\frac{9k_{3}^{2}-4k_{2}k_{4}}{16k_{2}^{5}} \end{array}$$

For further analysis, the phase term should be decomposed into its Taylor series of $f_{r}$ at $f_{r}=0$, then reorganized as:

(14) $$\begin{array}{l} \psi_{e}\left(f_{r},f_{a}\right)=\psi_{0}\left(f_{a}\right)+\psi_{1}\left(f_{a}\right)f_{r}+\psi_{2}\left(f_{a}\right)f_{r}^{2}\\ \;+\psi_{3}\left(f_{a}\right)f_{r}^{3}+\psi_{4}\left(f_{a}\right)f_{r}^{4}+\psi_{a}\left(f_{a}\right)+\psi_{r}\left(f_{r}\right) \end{array}$$

where:

(15) $$\begin{array}{l} \psi_{0}\left(f_{a}\right)=-2\pi \frac{f_{c}}{c}\left(R_{t}+R_{r0}\right)\\ \;+\pi \frac{f_{c}}{c}\left(A_{1}k_{1}^{2}-\frac{2}{3}A_{2}k_{1}^{3}+\frac{1}{2}A_{3}k_{1}^{4}\right)\\ \;+\pi \frac{c}{f_{c}}\left(A_{1}-2A_{2}k_{1}-3A_{3}k_{1}^{2}\right)f_{a}^{2}\\ \;+\pi \frac{c^{2}}{f_{c}^{2}}\left(-\frac{2}{3}A_{2}+2A_{3}k_{1}\right)f_{a}^{3}+\pi \frac{c^{3}}{f_{c}^{3}}\frac{1}{2}A_{3}f_{a}^{4} \end{array}$$

(16) $$\begin{array}{l} \psi_{1}\left(f_{a}\right)=\pi \frac{c}{f_{c}^{2}}\left(A_{1}-2A_{2}k_{1}+3A_{3}k_{1}^{2}\right)f_{a}^{2}\\ \;+\pi \frac{c^{2}}{f_{c}^{3}}\left(\frac{4}{3}A_{2}-4A_{3}k_{1}\right)f_{a}^{3}-\pi \frac{c^{3}}{f_{c}^{4}}\left(\frac{3}{2}A_{3}\right)f_{a}^{4} \end{array}$$

(17) $$\begin{array}{l} \psi_{2}\left(f_{a}\right)=-\pi \frac{1}{\gamma }+\pi \frac{c}{f_{c}^{3}}\left(A_{1}-2A_{2}k_{1}+3A_{3}k_{1}^{2}\right)f_{a}^{2}\\ \;+\pi \frac{c^{2}}{f_{c}^{4}}\left(-2A_{2}+6A_{3}k_{1}\right)f_{a}^{3}+\pi \frac{c^{3}}{f_{c}^{5}}3A_{3}f_{a}^{4} \end{array}$$

(18) $$\begin{array}{l} \psi_{3}\left(f_{a}\right)=\pi \frac{c}{f_{c}^{4}}\left(-A_{1}+2A_{2}k_{1}-3A_{3}k_{1}^{2}\right)f_{a}^{2}\\ \;+\pi \frac{c^{2}}{f_{c}^{5}}\left(\frac{8}{3}A_{2}-8A_{3}k_{1}\right)f_{a}^{3}-\pi \frac{c^{3}}{f_{c}^{6}}5A_{3}f_{a}^{4} \end{array}$$

(19) $$\begin{array}{l} \psi_{4}\left(f_{a}\right)=\pi \frac{c}{f_{c}^{5}}\left(A_{1}-2A_{2}k_{1}+3A_{3}k_{1}^{2}\right)f_{a}^{2}\\ \;+\pi \frac{c^{2}}{f_{c}^{6}}\left(-\frac{10}{3}A_{2}+10A_{3}k_{1}\right)f_{a}^{3}+\pi \frac{c^{3}}{f_{c}^{7}}\frac{15}{2}A_{3}f_{a}^{4} \end{array}$$

(20) $$\psi_{a}\left(f_{a}\right)=2\pi \left(A_{1}k_{1}-A_{2}k_{1}^{2}+A_{3}k_{1}^{3}\right)f_{a}$$

(21) $$\psi_{r}\left(f_{r}\right)=-\frac{\pi }{c}\left(2\left(R_{t}+R_{r0}\right)+A_{1}k_{1}^{2}-\frac{2}{3}A_{2}k_{1}^{3}+\frac{1}{2}A_{3}k_{1}^{4}\right)f_{r}$$

The azimuth compression term $\psi_{0}\left(f_{a}\right)$ only depends on $f_{a}$; thus, it can be compensated in the range time azimuth frequency domain. $\psi_{1}\left(f_{a}\right)$ represents the residual RCM after LRWC. The first term in $\psi_{2}\left(f_{a}\right)$ denotes the range modulation while the others are the SRC terms. $\psi_{3}\left(f_{a}\right)$ and $\psi_{4}\left(f_{a}\right)$ are high-order range-azimuth coupling terms. $\psi_{0}\left(f_{a}\right)$ and $\psi_{r}\left(f_{r}\right)$ represent the range and azimuth positions of target P after LRWC, respectively.

## 4. Imaging Algorithm for MBFL-SAR

In the aforementioned derivation, Equation (16) represents the phase of the 2-D frequency spectrum after LRWC. Then, the residual RCM, SRC, and high-order coupling terms are compensated in 2-D frequency domain, the function can be expressed as:

(22) $$H_{2}\left(f_{r},f_{a}\right)=\exp\left[-j\left(\psi_{1}f_{r}+\psi_{2}f_{r}^{2}+\psi_{3}f_{r}^{3}+\psi_{4}f_{r}^{4}\right)\right]$$

Transforming the result by range IFFT, the azimuth compression is completed by multiplying the function given as:

(23) $$H_{3}\left(t_{r},f_{a}\right)=\exp\left(-j\psi_{0}\right)$$

Finally, the 2-D focused SAR image can be obtained after the azimuth IFFT operation, which can be written as:

(24) $$s_{r5}\left(t_{r},t_{a}\right)=\mathrm{sinc}\left[B_{r}\left(t_{r}-\frac{R_{\mathrm{new}}}{c}\right)\right]\mathrm{sinc}\left[B_{a}\left(t_{a}-\frac{y_{\mathrm{new}}}{v_{x}}\right)\right]$$

where $R_{\mathrm{new}}=R_{t}+R_{r0}+\frac{1}{2}\left(A_{1}k_{1}^{2}-\frac{2}{3}A_{2}k_{1}^{3}+\frac{1}{2}A_{3}k_{1}^{4}\right)$ and $y_{\mathrm{new}}=A_{1}k_{1}-A_{2}k_{1}^{2}+A_{3}k_{1}^{3}$.

The compensation function sets the center point as the reference target point with an acceptable error, which will be discussed in the next section.

**Figure 2:**
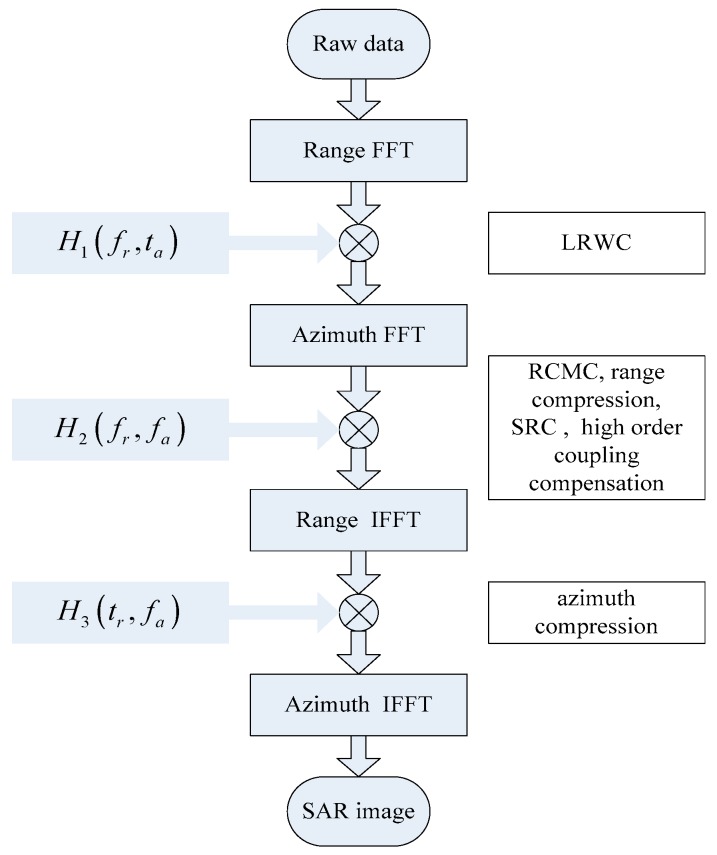
Block diagram of proposed imaging algorithm.

To summarize the operations above, the block scheme of the proposed BFL-SAR imaging algorithm is shown in Figure 2.

## 5. Simulations Results and Error Analysis

### 5.1. Simulations and Results

To demonstrate the efficiency of this algorithm, a simulation is presented in this section. The experiments are carried out in this section with the parameters shown in Table 1.

**Figure 3:**
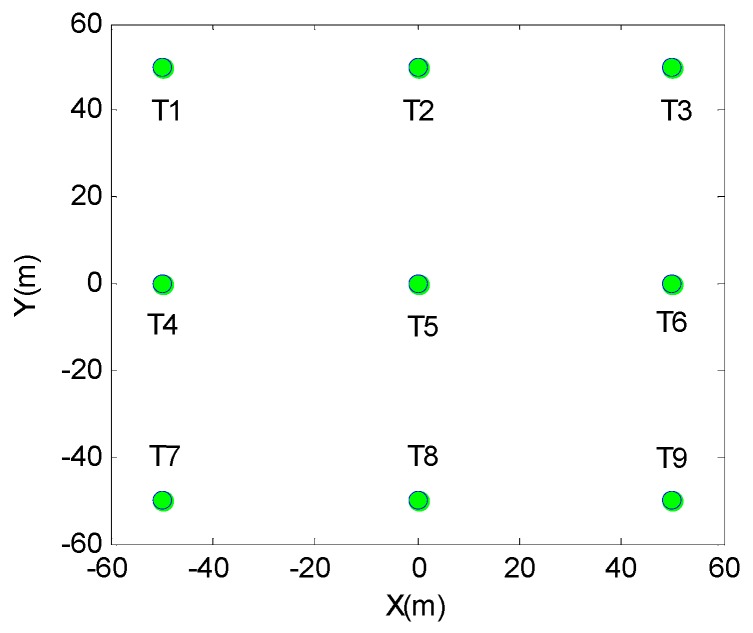
Scattering target positions.

The positions of these 3×3 scattering targets are shown in Figure 3. In the simulation, we set point target T5 as the reference center point. Point targets T4, T5, and T6 are located in the same azimuth direction and in different range directions, which means they have the same instantaneous Doppler frequency and different range cells. Point targets T2, T5, and T8 are located indifferent azimuth directions and in the same range direction, which means they have different instantaneous Doppler frequencies and the same range cell. 

**Figure 4:**
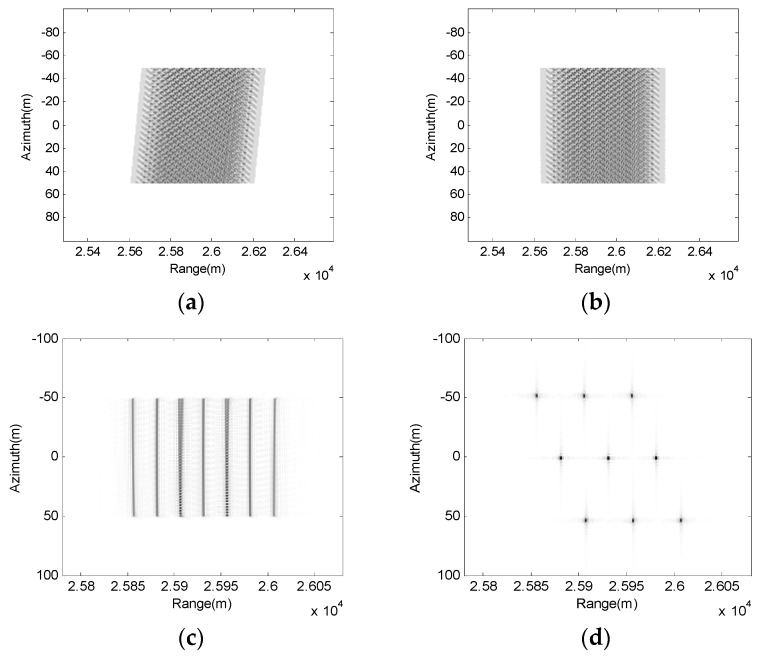
Interim simulation results using the proposed method. (**a**) Raw data; (**b**) After LRWC; (**c**) After range compression, RCMC, SRC, and high-order coupling compensation; (**d**) After azimuth compression.

**Figure 5:**
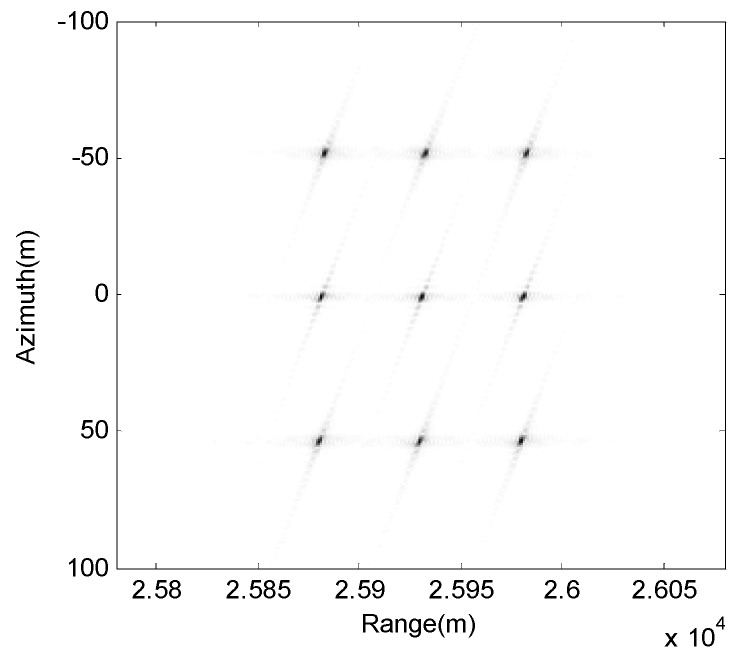
Imaging result after geometry correction.

Figure 4 shows the point target spectrum and interim simulation results using the proposed method. Figure 4a indicates the raw data from which we can see the skew due to the range azimuth coupling. Figure 4b shows the data after LRWC. The data is flattened, thus, the following steps can be performed easier. Figure 4c depicts the results after range compression, RCMC, SRC, and high-order coupling compensation, where each point target response is focused in the same range cell. Figure 4d shows the results of these 3×3 scattering targets after azimuth compression, and we can find the geometric distortion in the image due to the LRWC operation. The imaging result after geometric distortion correction is shown in Figure 5.

### 5.2. Error Analysis

In this paper, the main errors come from the following aspects: approximation of the slant range $R\left(t_{a}\right)$, approximation of the 2-D frequency spectrum derivation, the Taylor expansion of the 2-D frequency spectrum, and approximation of the compensation function. Here, we analyze the error to prove the effectiveness of this algorithm.

Firstly, the equivalence slant range is kept up to the fourth-order Taylor series expansion of $R\left(t_{a}\right)$, where the higher-order terms are ignored. The ideal one is shown in Equation (2) and the error can be expressed as:

(25) $$\Delta R\left(t_{a}\right)=R_{r}\left(t_{a}\right)-\left[R_{r0}+\left(k_{1c}+k_{1}\right)t_{a}+k_{2}t_{a}^{2}+k_{3}t_{a}^{3}+k_{4}t_{a}^{4}\right]$$

**Figure 6:**
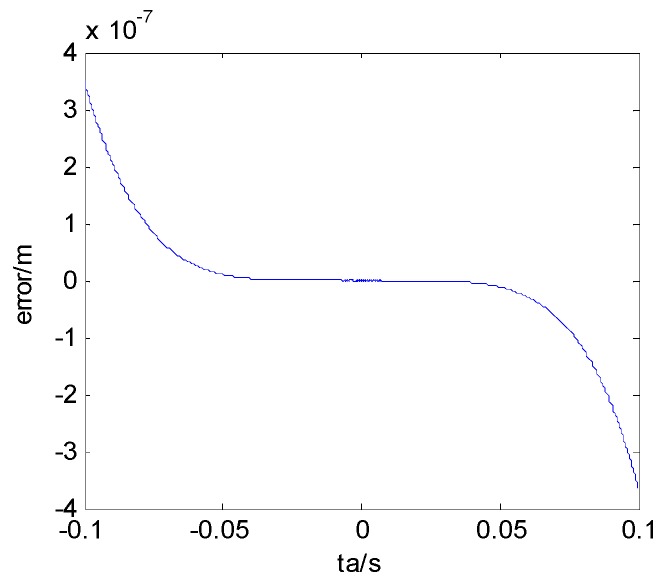
Slant range approximation error of high orders.

Taking the scene center point as the reference point, the parameters are listed in Table 1, and the results are shown in Figure 6.

The maximum range approximation error is $3\times 10^{-7}$m in Figure 6, which contributes to the phase error in Equation (5) as:

(26) $$\Delta \psi \left(f_{r},t_{a}\right)=-2\pi \frac{\left(f_{c}+f_{r}\right)}{c}\Delta R\left(t_{a}\right)$$

Using the simulation parameters into Equation (1), the phase error absolute value of our approximation is about $6.3\times 10^{-5}\;\mathrm{rad}$. It meets the demand of $\Delta \psi \leq \frac{\pi }{4}$.

**Figure 7:**
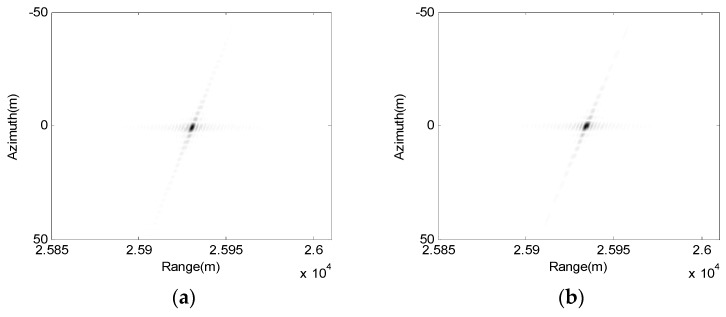
Focusing results of the point target using different 2-D spectra. (**a**) Series reversion; and (**b**) Numerical calculation.

Secondly, the approximation of the 2-D frequency spectrum by the method of POSP and series reversion introduces the error. This error partly depends on the range approximation, namely, it is related to the first error. Another aspect is the remaining Taylor expansion orders of $f_{r}$. In this algorithm, the 2-D frequency spectrum expansion of $f_{r}$ ignores the terms higher than fourth-order. To analyze this error, the method of numerical calculation is considered as a comparison, which can obtain the accurate 2-D frequency spectrum. Figure 7 shows the imaging results of point targets using the approximation of 2-D frequency spectrum derived in this paper and the method of numerical calculation, respectively.

**Figure 8:**
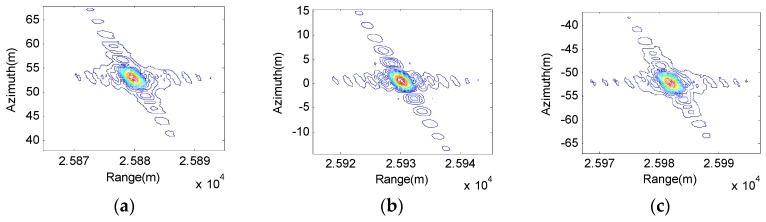
Focusing performance of proposed improved RDA: (**a**) Target 1; (**b**) Target 5; and (**c**) Target 9.

The last one, also the greatest cause of algorithm accuracy, is the approximation of k-coefficients which uses $y_{\mathrm{pc}}$ instead of $y_{p}$. Thus, we compare subimages of the center point T5 and edge points T1 and T9, which can be shown in Figure 8.

**Figure 9:**
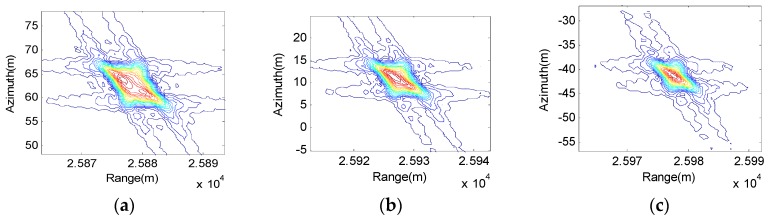
Focusing performance of traditional RDA: (**a**) Target 1; (**b**) Target 5; (**c**) Target 9.

From Figure 8, we can find that the focusing performance of the center point is better than the edge point. However, the edge point can still focus well with our algorithm. To make a clearer performance comparison between the proposed algorithm and the traditional RDA, the imaging result obtained by traditional RDA is shown Figure 9.

Comparing the results, it is easy to find that the proposed algorithm generally offers better focusing capabilities than the traditional RDA. Analyzing the data in Figure 8 and Figure 9, the resolution ρ, peak sidelobe ratio (PSLR) and integrated sidelobe ratio (ISLR) are used to evaluate the focusing performance. T1 and T5 are chosen for analysis. The results are listed in Table 2. We can find that higher resolution and the theoretical PSLR and ISLR are obtained by the proposed algorithm.

## 6. Conclusions

In this paper, an imaging algorithm for a fixed-transmitter BFL-SAR is proposed, which takes high-order Taylor series expansion terms of the slant range into consideration. Firstly, the signal model of BFL-SAR with fixed transmitter is built. Secondly, the high-precision 2-D frequency spectrum is derived by the method of series reversion. This method uses the fourth-order Taylor expansion of the slant range after LRWC operation. Then, the residual RCM, SRC, and high-order coupling terms are compensated in the 2-D frequency domain. Finally, a SAR image can be obtained by 2-D compression. The simulation results and error analysis prove the feasibility and effectiveness of the proposed imaging algorithm and verify the correctness of the theory analysis.

## Figures and Tables

**Table 1:** Simulation parameters.

Parameters	Values	Parameters	Values
Velocity (m/s)	(0, 1000, −30)	Carrier frequency (GHz)	10
Acceleration (m/s^2^)	(0, −1, −5)	Pulse duration (μm)	1.5
Transmitter position	(−20,000, 3000, 2000)	Bandwidth (MHz)	150
Scene center	(0, 3000, 0)	Range samples	2048
Initial receiver position	(0, 0, 5000)	Azimuth samples	2048
Synthetic aperture length (m)	200		

**Table 2:** Characteristic parameters of center point and edge point.

	Range			Azimuth		
	ρ (m)	PSLR (dB)	ISLR (dB)	ρ (m)	PSLR (dB)	ISLR (dB)
Proposed improved RDA						
Edge point T1	3.5	−13.5	−10.8	5.3	−13.5	−10.2
Center point T5	3.5	−13.1	−10.0	5.1	−13.5	−10.2
Traditional RDA						
Edge point T1	6.5	−20.8	−22.2	10.5	−22.7	−20.3
Center point T5	5.5	−20.7	−19.9	8.8	−19.5	−17.2
